# Integration of polygenic and gut metagenomic risk prediction for common diseases

**DOI:** 10.1038/s43587-024-00590-7

**Published:** 2024-03-25

**Authors:** Yang Liu, Scott C. Ritchie, Shu Mei Teo, Matti O. Ruuskanen, Oleg Kambur, Qiyun Zhu, Jon Sanders, Yoshiki Vázquez-Baeza, Karin Verspoor, Pekka Jousilahti, Leo Lahti, Teemu Niiranen, Veikko Salomaa, Aki S. Havulinna, Rob Knight, Guillaume Méric, Michael Inouye

**Affiliations:** 1 Cambridge Baker Systems Genomics Initiative, Department of Public Health and Primary Care, University of Cambridge, Cambridge, UK; 2 Cambridge Baker Systems Genomics Initiative, Baker Heart and Diabetes Institute, Melbourne, Victoria Australia; 3 Department of Clinical Pathology, Melbourne Medical School, University of Melbourne, Melbourne, Victoria Australia; 4 Victor Phillip Dahdaleh Heart and Lung Research Institute, University of Cambridge, Cambridge, UK; 5 British Heart Foundation Cardiovascular Epidemiology Unit, Department of Public Health and Primary Care, University of Cambridge, Cambridge, UK; 6 British Heart Foundation Cambridge Centre of Research Excellence, School of Clinical Medicine, University of Cambridge, Cambridge, UK; 7 Health Data Research UK Cambridge, Wellcome Genome Campus and University of Cambridge, Cambridge, UK; 8 Centre for Youth Mental Health, University of Melbourne, Melbourne, Victoria Australia; 9 Department of Public Health and Welfare, Finnish Institute for Health and Welfare, Helsinki, Finland; 10 Department of Computing, University of Turku, Turku, Finland; 11 School of Life Sciences, Arizona State University, Tempe, AZ USA; 12 Biodesign Center for Fundamental and Applied Microbiomics, Arizona State University, Tempe, AZ USA; 13 Department of Ecology and Evolutionary Biology, Cornell University, Ithaca, NY USA; 14 Center for Microbiome Innovation, Jacobs School of Engineering, University of California San Diego, La Jolla, CA USA; 15 School of Computing Technologies, RMIT University, Melbourne, Victoria Australia; 16 School of Computing and Information Systems, University of Melbourne, Melbourne, Victoria Australia; 17 Division of Medicine, Turku University Hospital and University of Turku, Turku, Finland; 18 Institute for Molecular Medicine Finland, FIMM-HiLIFE, University of Helsinki, Helsinki, Finland; 19 Department of Computer Science and Engineering, University of California San Diego, La Jolla, CA USA; 20 Department of Pediatrics, School of Medicine, University of California San Diego, La Jolla, CA USA; 21 Central Clinical School, Monash University, Melbourne, Victoria Australia; 22 Department of Cardiometabolic Health, University of Melbourne, Melbourne, Victoria Australia; 23 Department of Cardiovascular Research, Translation and Implementation, La Trobe University, Melbourne, Victoria Australia; 24 Department of Medical Sciences, Molecular Epidemiology, Uppsala University, Uppsala, Sweden; 25 The Alan Turing Institute, London, UK

**Keywords:** Predictive markers, Diseases

## Abstract

Multiomics has shown promise in noninvasive risk profiling and early detection of various common diseases. In the present study, in a prospective population-based cohort with ~18 years of e-health record follow-up, we investigated the incremental and combined value of genomic and gut metagenomic risk assessment compared with conventional risk factors for predicting incident coronary artery disease (CAD), type 2 diabetes (T2D), Alzheimer disease and prostate cancer. We found that polygenic risk scores (PRSs) improved prediction over conventional risk factors for all diseases. Gut microbiome scores improved predictive capacity over baseline age for CAD, T2D and prostate cancer. Integrated risk models of PRSs, gut microbiome scores and conventional risk factors achieved the highest predictive performance for all diseases studied compared with models based on conventional risk factors alone. The present study demonstrates that integrated PRSs and gut metagenomic risk models improve the predictive value over conventional risk factors for common chronic diseases.

## Abstract (web summary)

In a longitudinal population-based cohort, Liu et al. demonstrate that integrating polygenic risk scores and the gut microbiome improved prediction, over traditional risk factors, for heart disease, diabetes, Alzheimer disease and prostate cancer.

## Main

Multiomic technologies have uncovered potential biomarkers for various common age-related diseases, including cardiovascular disease, diabetes, liver disease, dementia and cancer^1–6^. Although conventional risk prediction typically relies on demographic (for example, age or sex), anthropomorphic (for example, body mass index (BMI)), lifestyle factors and disease-specific clinical laboratory measurements (for example, blood pressure (BP), non-high-density lipoprotein (HDL)-cholesterol, mammographic density, creatinine, glycated hemoglobin (HbA1c)), the recent emergence of multiomics means that it is now possible to measure and integrate whole classes of biomolecular and cellular factors for the purposes of building multiomic risk scores.

PRSs, a quantitative measure of genetic predisposition for a phenotype, have demonstrated validity and potential clinical utility in risk prediction for various common diseases^7–10^, for example, in cardiovascular disease^11–14^, cancers^15,16^, diabetes mellitus^17–19^ and ankylosing spondylitis^20^. Given the potential of a genome-wide genotyping array as a one-time, relatively inexpensive assay from which hundreds of PRSs can be calculated, PRSs are being assessed in clinical studies for healthcare systems around the world^9,11,21^.

The gut microbiota (the collection of microorganisms inhabiting the human gastrointestinal tract) has also been shown to have a role in many common diseases^22–24^. Gut microbial signatures have been associated with mortality and incident diseases in the general population, such as type 2 diabetes (T2D) and liver and respiratory diseases^4,25–29^, suggesting the potential of the gut microbiome in disease risk prediction. Notably, although genome-wide association studies have revealed the human genetic basis of the gut microbiome^30–32^, it is apparent that the heritability of the gut microbiome is relatively low and cross-generational familial microbiome similarity is largely associated with cohabitation^33–35^.

Given that they are based on robust scalable technologies, use noninvasive sampling and have been applied in numerous disease risk prediction studies, PRSs and the gut microbiome comprise promising components of potential future multiomic risk prediction^36,37^. It has been previously shown that the gut microbiome and host genetics independently contribute to cross-sectional prediction of host metabolic traits, with improved prediction performance by combining genetics and microbiome over modeling based on host genetics and environmental factors^38^. However, many previous microbiome studies of disease have retrospective case–control designs, which are susceptible to various selection biases (for example, ascertainment, geographical, demographic biases) as well as technical differences such as sample storage^39,40^. Prospective studies minimize the risk of many of these biases and enable risk prediction of future disease. Furthermore, the extent to which host genetics and microbiome can jointly predict future risk of common diseases, including their additive value to baseline age and other conventional risk factors, remains unclear.

In the present study, we investigate the predictive capacity of PRSs, the gut microbiome and conventional risk factors for multiple incident common diseases using a population-based prospective cohort. We focus on diseases for which there is prior evidence of substantial predictive capacity for PRSs and the human gut microbiome, that is, coronary artery disease (CAD)^12,41^, T2D^26,42^, Alzheimer disease (AD)^43,44^ and prostate cancer^45,46^. We utilized the population-based, multiomic FINRISK 2002 cohort^47^ to assess the individual and combined performance of PRSs, gut microbiome scores and conventional risk factors to incident disease. Finally, we generated and validated multiomic predictive models for each disease and have made these available to the research community.

## Results

For those in FINRISK 2002 with imputed genotypes and gut metagenomic sequencing, there were 333 incident cases of CAD, 579 of T2D, 273 of AD and 141 of prostate cancer over a median follow-up of 17.8 years through electronic health records (EHRs). Characteristics of the study sample of FINRISK 2002 cohort for each disease are given in Table 1. For CAD, T2D and AD, baseline clinical risk factors were significantly different between incident cases and non-cases with the exception of smoking for T2D, and sex, diastolic BP (DBP) and HDL for AD. We detected significant differences between case and non-case groups in baseline age and smoking for prostate cancer.

**Table 1:** Characteristics of participant risk factors for the diseases studied

	Cases	Non-cases	*P* value
**CAD**	*n* = 333	*n* = 4,760	
Male, *n* (%)	225 (67.57)	2,015 (42.33)	3.62 × 10^−19^
Baseline age (years)	56.81 ± 9.74	47.55 ± 12.40	4.58 × 10^−39^
BMI (kg m^−2^)	27.91 ± 3.96	26.46 ± 4.24	4.27 × 10^−11^
SBP (mmHg)	144.90 ± 20.07	134.10 ± 19.36	3.36 × 10^−23^
Total cholesterol (mmol l^−1^)	6.02 ± 1.09	5.58 ± 1.05	9.57 × 10^−13^
HDL (mmol l^−1^)	1.37 ± 0.39	1.53 ± 0.41	1.84 × 10^−14^
Smoking, *n* (%)	106 (31.83)	1,165 (24.47)	3.87 × 10^−3^
Exercise, *n* (%)	52 (15.62)	1,182 (24.83)	9.03 × 10^−5^
Prevalent diabetes, *n* (%)	26 (7.81)	137 (2.88)	1.56 × 10^−5^
Family history, *n* (%)	130 (39.04)	1,142 (23.99)	4.25 × 10^−9^
**T2D**	*n* = 579	*n* = 4,718	
Male, *n* (%)	306 (52.85)	2,114 (44.81)	2.84 × 10^−4^
Baseline age (years)	53.26 ± 10.57	48.37 ± 12.89	1.14 × 10^−18^
BMI (kg m^−2^)	29.98 ± 4.18	26.13 ± 3.99	1.27 × 10^−88^
SBP (mmHg)	142.67 ± 20.81	134.50 ± 19.65	4.67 × 10^−21^
Total cholesterol (mmol l^−1^)	5.84 ± 1.20	5.58 ± 1.04	2.43 × 10^−6^
HDL (mmol l^−1^)	1.35 ± 0.35	1.54 ± 0.41	9.72 × 10^−32^
Triglyceride (mmol l^−1^)	1.91 ± 1.29	1.32 ± 0.83	8.41 × 10^−6^
Smoking, *n* (%)	160 (27.63)	1,155 (24.48)	0.103
Exercise, *n* (%)	82 (14.16)	1,168 (24.76)	3.80 × 10^−9^
Family history, *n* (%)	251 (43.35)	1,159 (24.57)	2.57 × 10^−20^
**AD**	*n* = 273	*n* = 5,074	
Male, *n* (%)	128 (46.89)	2,349 (46.29)	0.852
Baseline age (years)	64.29 ± 6.52	48.21 ± 12.46	1.07 × 10^−93^
BMI (kg m^−2^)	28.08 ± 4.05	26.59 ± 4.24	1.38 × 10^−9^
SBP (mmHg)	144.82 ± 20.59	135.01 ± 19.90	5.60 × 10^−16^
DBP (mmHg)	79.63 ± 10.08	79.14 ± 11.17	0.489
Total cholesterol (mmol l^−1^)	5.84 ± 1.12	5.57 ± 1.05	1.07 × 10^−4^
HDL (mmol l^−1^)	1.50 ± 0.45	1.51 ± 0.41	0.304
Alcohol consumption (g per week)	62.63 ± 138.15	82.76 ± 123.58	1.77 × 10^−8^
Smoking, *n* (%)	46 (16.85)	1,279 (25.21)	1.50 × 10^−3^
Exercise, *n* (%)	44 (16.12)	1,219 (24.02)	2.62 × 10^−3^
Prevalent T2D, *n* (%)	18 (6.59)	128 (2.52)	4.03 × 10^−4^
Prevalent stroke, *n* (%)	13 (4.76)	100 (1.97)	7.20 × 10^−3^
Prevalent psychiatric disorders, *n* (%)	12 (4.40)	121 (2.38)	0.045
**Prostate cancer**	*n* = 141	*n* = 2,323	
Baseline age (years)	59.79 ± 7.66	49.39 ± 12.62	1.79 × 10^−22^
BMI (kg m^−2^)	27.45 ± 3.03	27.07 ± 3.81	0.086
Alcohol consumption (g per week)	113.70 ± 147.06	123.40 ± 152.37	0.819
Smoking, *n* (%)	23 (16.31)	716 (30.82)	1.97 × 10^−4^
Exercise, *n* (%)	34 (24.11)	607 (26.13)	0.693
Family history, *n* (%)	62 (43.97)	794 (34.18)	0.022

Numerical variables are shown as mean ± s.d. Categorical variables are shown as the number of individuals and percentage of their respective disease status group. *P* values of two-sided Mann–Whitney *U*-test and Fisher’s exact test are reported for numerical and categorical variables, respectively.

### PRSs and conventional risk factors

Previously validated PRSs for CAD^12^ (PGS000018), T2D^42^ (PGS000036), AD^43^ (PGS000334) and prostate cancer^45^ (PGS000662) were obtained from the Polygenic Score Catalog^48^ (Methods). Cox regression models were used to assess the predictive performance of PRSs and disease-specific conventional risk factors for incident diseases.

**Fig. 1:**
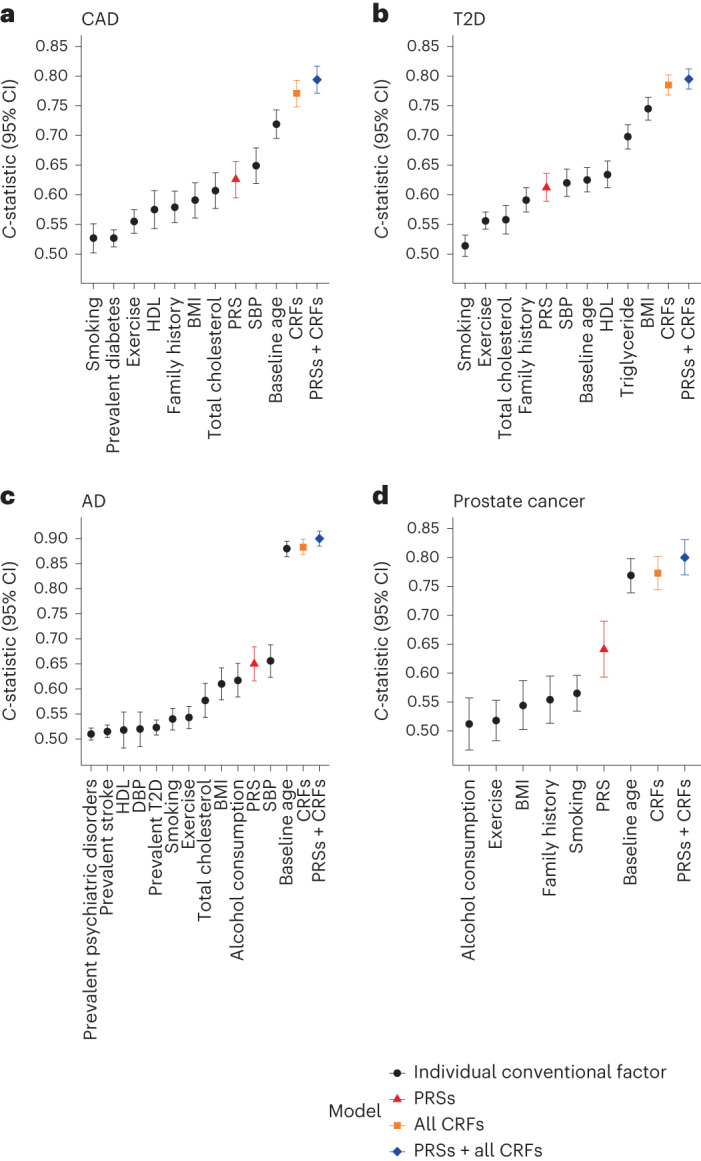
Prediction performance of PRSs and conventional risk factors. **a**–**d**, *C*-statistics of Cox models of disease-specific CRFs and PRSs for incident CAD (*n* = 5,093) (**a**), T2D (*n* = 5,297) (**b**), AD (*n* = 5,347) (**c**) and prostate cancer (*n* = 2,464) (**d**). CRFs and PRSs are modeled individually and jointly. Cox proportional hazard models for CAD, T2D and AD are stratified by sex. The *C*-statistics are depicted alongside their 95% CIs as dots and error bars. Source data

We first assessed prediction performance of PRSs and conventional risk factors (Methods) individually for their respective incident diseases (Fig. 1). In sex-stratified (except for prostate cancer) Cox models of individual risk factors for incident CAD, AD and prostate cancer, baseline age had the highest concordance statistic (*C*-statistic) (0.719, 95% confidence interval (CI) 0.695–0.743; 0.880, 95% CI 0.864–0.895; and 0.769, 95% CI 0.739–0.798, respectively). For CAD and AD, systolic BP (SBP) was the second strongest individual factor by *C*-statistics (0.649, 95% CI 0.619–0.679 and 0.656, 95% CI 0.623–0.688, respectively), followed by comparable *C*-statistics for PRSs (0.626, 95% CI 0.595–0.656 and 0.650, 95% CI 0.616–0.684, respectively). For incident prostate cancer, the PRS was stronger than other individual conventional risk factors except baseline age with a *C*-statistic of 0.641 (95% CI 0.593–0.690). For incident T2D, the BMI had the strongest *C*-statistic (0.745, 95% CI 0.726–0.764) and the PRS had a *C*-statistic of 0.612 (95% CI 0.589–0.636), similar to the other conventional risk factors. The PRS alone achieved a higher *C*-statistic than family history for all diseases where this was available, including CAD, T2D and prostate cancer.

**Extended Data Fig. 1:**
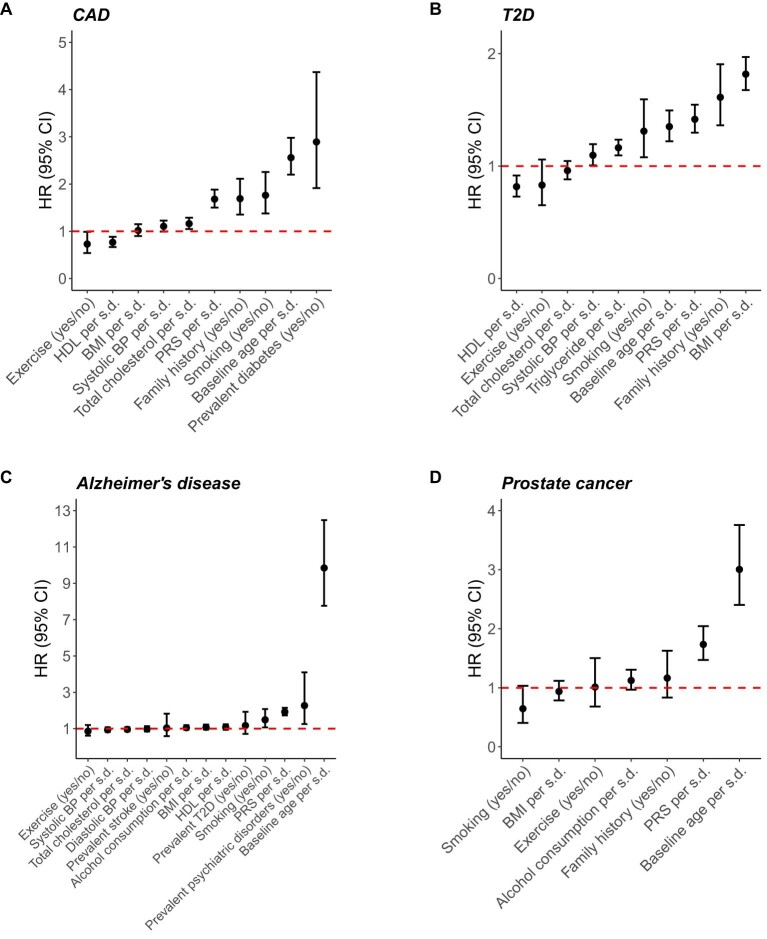
Significant associations between PRSs and incident diseases. Cox proportional hazards models of disease-specific PRSs and conventional risk factors are fit for (**a**) CAD (n = 5,093), (**b**) T2D (n = 5,297), (**c**) AD (n = 5,347) and (**d**) prostate cancer (n = 2,464). Cox models for CAD, T2D and AD are stratified by sex. Hazard ratios (HRs) of risk factors are depicted alongside their 95% confidence intervals (CIs) as dots and error bars. Source data

**Extended Data Fig. 2:**
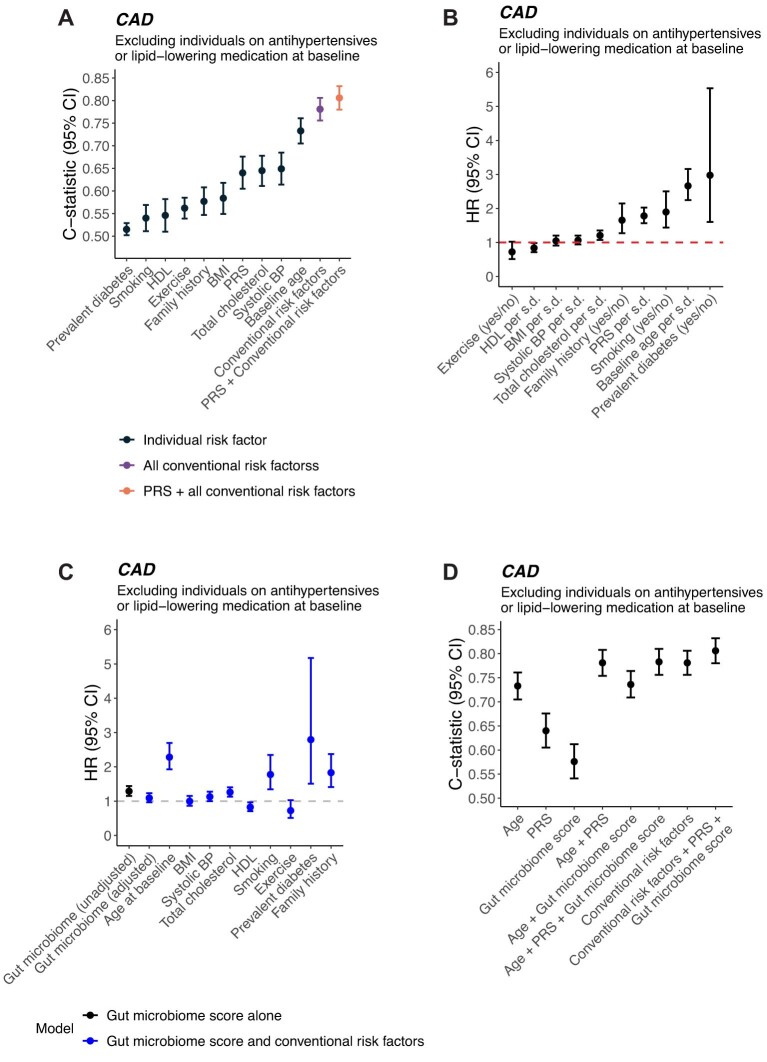
Sub-analysis of incident CAD in individuals who were not on antihypertensives and lipid-lowering medications at baseline (n = 4,293). In sex-stratified Cox models of PRS and conventional risk factors, (**a**) C-statistics and (**b**) hazard ratios (HRs) are depicted alongside their 95% confidence intervals (CIs) as dots and error bars. In sex-stratified Cox models of the gut microbiome score and conventional risk factors, (**c**) HRs of the gut microbiome score and conventional risk factors are depicted alongside their 95% CIs as dots and error bars. (**d**) In Cox models for integrative analysis, C-statistics and their 95% CIs of are presented as dots and error bars. Source data

In assessing the incremental gain in prediction of each PRS over its disease-specific conventional risk factors (Fig. 1), we found ∆*C*-indices of 0.023 for CAD (95% CI 0.013–0.034), 0.01 for T2D (95% CI 0.004–0.016), 0.017 for AD (95% CI 0.010–0.024) and 0.027 for prostate cancer (95% CI 0.009–0.047). As expected, all PRSs were significantly associated with their respective incident diseases after adjusting for disease-specific conventional risk factors, and baseline age remained the strongest predictor for CAD, AD and prostate cancer (Extended Data Fig. 1). We observed hazard ratios (HRs) per s.d. for PRS levels of 1.68 for CAD (95% CI 1.50–1.88, *P* = 2.25 × 10^−19^), 1.42 for T2D (95% CI 1.30–1.55, *P* = 6.48 × 10^−15^), 1.92 for AD (95% CI 1.73–2.15, *P* = 4.27 × 10^−32^) and 1.73 for prostate cancer (95% CI 1.47–2.04, *P* = 5.50 × 10^−11^). The effects of PRSs and family history were independent for incident CAD, T2D and prostate cancer, implying that the PRS and family history complement each other. As a subanalysis for CAD, we excluded individuals taking antihypertensives and lipid-lowering medications at baseline (Extended Data Fig. 2a,b), with the findings being consistent with the main analysis of all individuals.

**Extended Data Fig. 3:**
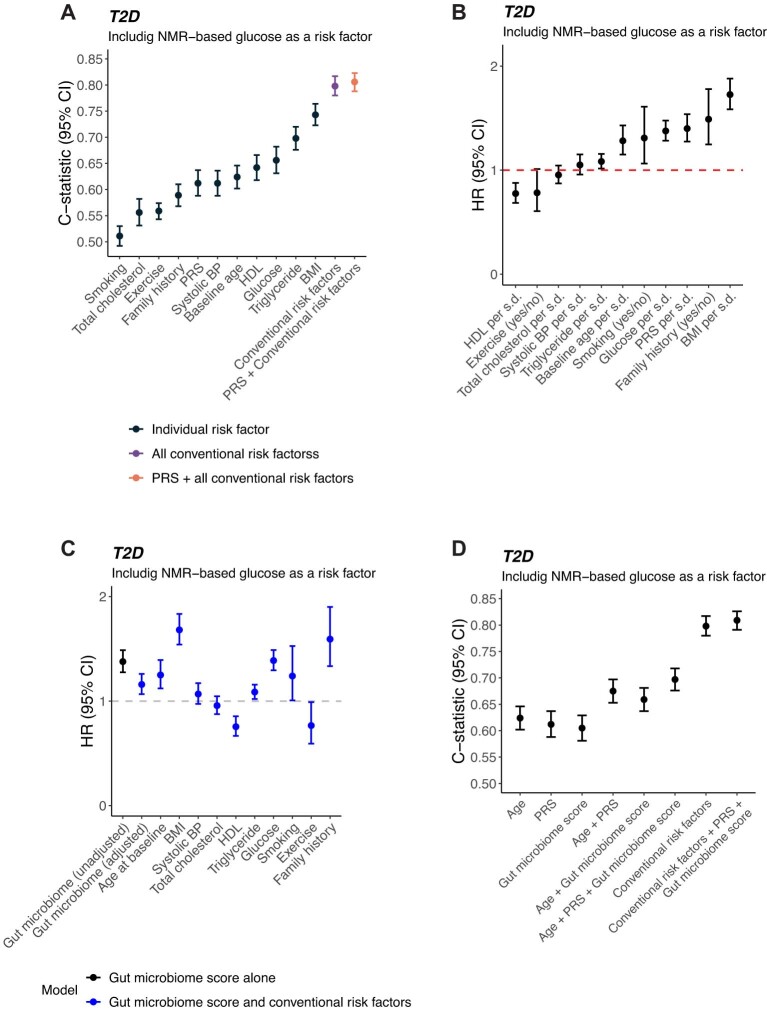
Sub-analysis of incident T2D (n = 4,911) using NMR-determined glucose as an additional risk factor in sex-stratified Cox models. In sex-stratified Cox models of PRS and conventional risk factors, (**a**) C-statistics and (**b**) hazard ratios (HRs) are depicted alongside their 95% confidence intervals (CIs) as dots and error bars. In sex-stratified Cox models of the gut microbiome score and conventional risk factors, (**c**) HRs of the gut microbiome score and conventional risk factors are depicted alongside their 95% CIs as dots and error bars. (**d**) In Cox models for integrative analysis, C-statistics and their 95% CIs of are presented as dots and error bars. Source data

For T2D, we performed a subanalysis using nuclear magnetic resonance (NMR)-determined glucose as an additional conventional risk factor (Extended Data Fig. 3a,b). In sex-stratified Cox models of individual risk factors, BMI again had the strongest *C*-statistic (0.743, 95% CI 0.723–0.764), whereas the PRS and glucose had *C*-statistics of 0.612 (95% CI 0.588–0.637) and 0.656 (95% CI 0.631–0.682), respectively. Adding the PRS increased the *C*-statistic over the model of conventional risk factors by 0.007 (95% CI 0.001–0.013). In the model combining PRSs and conventional risk factors, the PRS and glucose were both significantly associated with incident T2D with similar effect sizes (HR = 1.40 per s.d., 95% CI 1.27–1.54, *P* = 1.85 × 10^−12^ and HR = 1.38 per s.d., 95% CI 1.28–1.48, *P* = 5.95 × 10^−19^).

**Extended Data Fig. 4:**
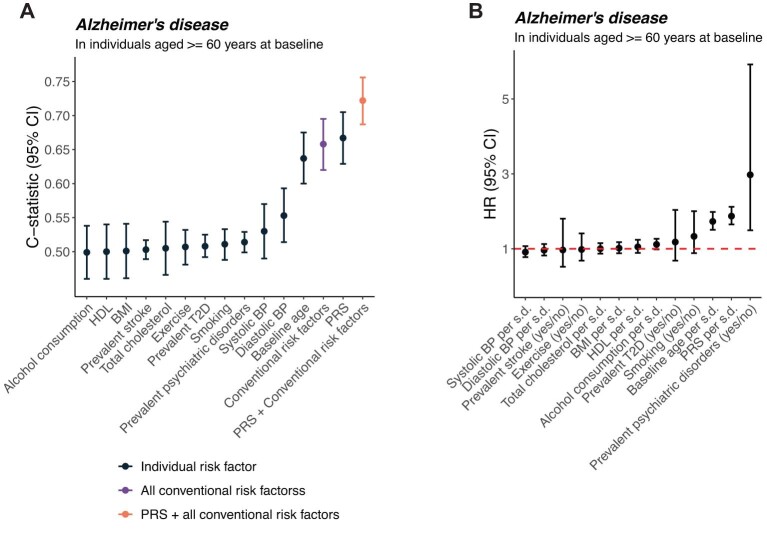
Sub-analysis of incident AD in participants aged 60 and above at baseline (n = 1,220) using sex-stratified Cox models of conventional risk factors and PRS. (**a**) C-statistics and (**b**) hazard ratios (HRs) are depicted as dots and their 95% confidence intervals (CIs) are depicted as error bars. Source data

In a subanalysis of AD in participants aged ≥60 years (Extended Data Fig. 4), the sex-stratified Cox model of the PRS alone with a *C*-statistic of 0.667 (95% CI 0.629–0.705) was greater than any individual conventional risk factor as well as the model combining all conventional factors. Adding the PRS improved the *C*-statistic over conventional risk factors by 0.064 (95% CI 0.036–0.096), leading to a model with a *C*-statistic of 0.722 (95% CI 0.687–0.756). Notably, in the model combining PRSs and all conventional risk factors of AD, the PRS was associated with an incident AD with an HR of 1.87 (95% CI 1.65–2.12, *P* = 8.95 × 10^−23^) per s.d., which was greater than that for baseline age (HR = 1.73 per s.d., 95% CI 1.51–1.98, *P* = 4.50 × 10^−15^).

### Gut microbiome and incident disease

In FINRISK 2002, the gut microbiome composition was determined by shallow shotgun metagenomic sequencing of baseline stool samples (Methods). To investigate the association between incident diseases and the overall variation in gut microbial communities, we performed Cox analyses on *α* and *β* diversity at the species level, adjusting for disease-specific conventional risk factors. The *α* diversity was estimated using the Shannon index, the Chao–Shannon index^49^, species richness and evenness. The Shannon index and the Chao–Shannon index were significantly negatively associated with incident T2D (HR 0.89 per s.d., 95% CI 0.82–0.96, *P* = 0.004 and HR 0.90 per s.d., 95% CI 0.82–0.98, *P* = 0.014, respectively), complementing the previously reported negative association between T2D and gut microbiome richness^50^; species richness was associated with incident prostate cancer (HR 1.23 per s.d., 95% CI 1.1–1.39, *P* = 4.20 × 10^−4^); no significant association was observed for incident CAD and AD (Supplementary Table 1). In the analysis of *β* diversity between samples using principal component analysis (PCA) of the Aitchison distance, incident T2D was associated with principal component (PC)2 (HR 0.94, 95% CI 0.91–0.96, *P* = 1.31 × 10^−5^) and PC5 (HR 1.04, 95% CI 1.00–1.08, *P* = 0.030). In comparison, using principal coordinate analysis based on the Bray–Curtis dissimilarity, incident T2D was associated with PC1 (HR 1.78, 95% CI 1.08–2.95, *P* = 0.024) and PC5 (HR 3.26, 95% CI 1.44–7.38, *P* = 0.005). No significant associations were observed for CAD, AD and prostate cancer.

**Extended Data Fig. 5:**
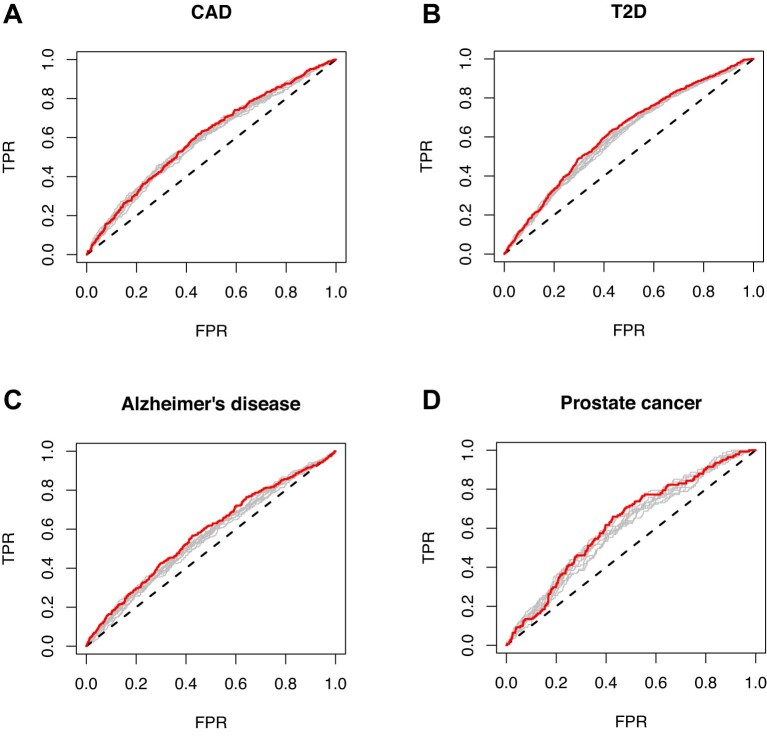
Cross-validated Ridge logistic regression models for incident (a) CAD, (b) T2D, (c) AD and (d) prostate cancer using gut microbiome composition. The ROC curve of the optimal cross-validated model is in red and curves of other models are in grey. Source data

To investigate the predictive capacity of gut microbial taxa for incident diseases, we focused on 235 species-level taxonomic groups after excluding rare and less prevalent taxa (Methods). In developing prediction models with taxa abundance at species levels, we utilized ridge logistic regression with 10× three-fold stratified cross-validation (Methods). The average cross-validated area under the receiver operating characteristic curve (AUROC) of the models was 0.597 (range 0.588–0.605) for CAD, 0.610 (0.599–0.624) for T2D, 0.564 (0.552–0.582) for AD and 0.613 (0.595–0.626) for prostate cancer (Extended Data Fig. 5). In subanalyses, similar AUROCs of cross-validated models were achieved for CAD (mean 0.587, range 0.552–0.609) and T2D (mean 0.604, range 0.589–0.614), whereas the gut microbiome was not predictive of AD in participants aged ≥60 years at baseline.

**Fig. 2:**
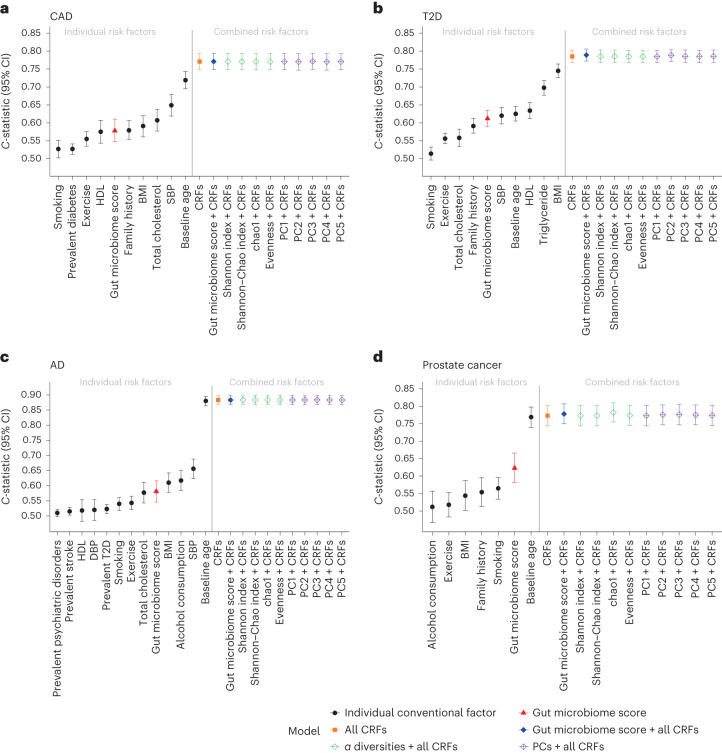
Prediction performance of gut microbial features and conventional risk factors. **a**–**d**, *C*-statistics of Cox models of disease-specific CFRs and gut microbial features for incident CAD (*n* = 5,093) (**a**), T2D (*n* = 5,297) (**b**), AD (*n* = 5,347) (**c**) and prostate cancer (*n* = 2,464) (**d**). CRFs and gut microbiome scores are modeled individually and jointly. The *α* diversities and five PCs of CLR abundance are modeled with adjustment for all disease-specific CRFs. Cox proportional hazard models for CAD, T2D and AD are stratified by sex. The *C*-statistics are depicted alongside their 95% CIs as dots and error bars. Source data

**Extended Data Fig. 6:**
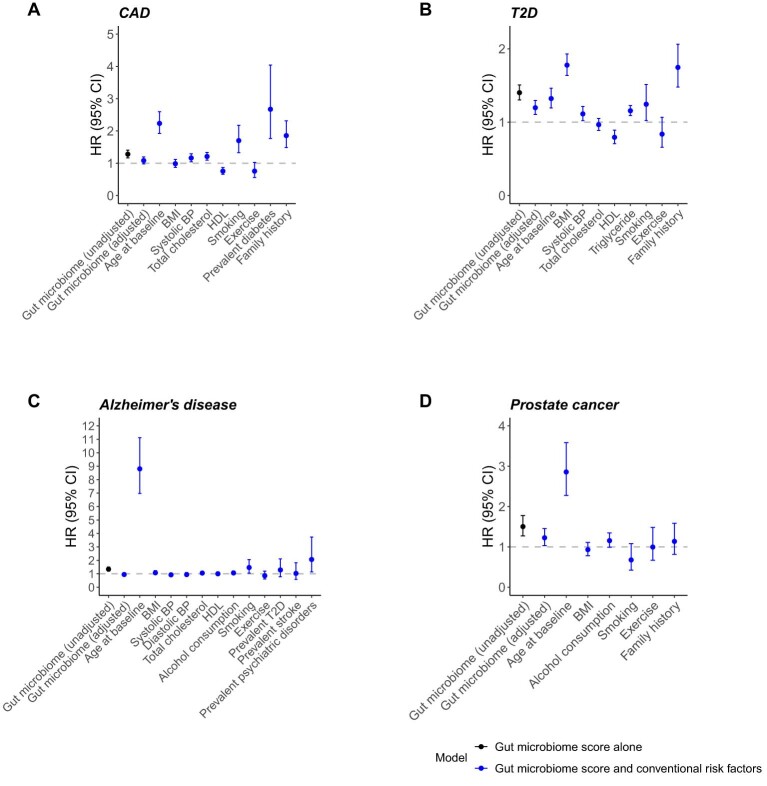
Cox proportional hazards models of disease-specific gut microbiome scores and conventional risk factors for (a) incident CAD (n = 5,093), (b) T2D (n = 5,297), (c) AD (n = 5,347) and (d) prostate cancer (n = 2,464). The gut microbiome score is modelled individually and in combination with conventional risk factors. Cox models for CAD, T2D and AD are stratified by sex. Hazard ratios (HRs) of risk factors are depicted alongside their 95% confidence intervals (CIs) as dots and error bars. Source data

In sex-stratified (except for prostate cancer) Cox regression models, the gut microbiome score alone was significantly associated with all incident diseases (Extended Data Fig. 6), with HRs of 1.28 (95% CI 1.17–1.41, *P* = 2.29 × 10^−7^), 1.40 (95% CI 1.30–1.51, *P* = 7.45 × 10^−20^), 1.34 (95% CI 1.20–1.50, *P* = 2.09 × 10^−7^) and 1.50 (95% CI 1.27–1.78, *P* = 1.66 × 10^−6^) per s.d. for incident CAD, T2D, AD and prostate cancer, respectively. For CAD and T2D, the gut microbiome scores individually showed similar performance in *C*-statistics compared with a few conventional risk factors including family history (0.578, 95% CI 0.547–0.61 and 0.612, 95% CI 0.590–0.635, respectively; Fig. 2). For AD, the gut microbiome score achieved a higher *C*-statistic (0.581, 95% CI 0.546–0.616) than BP, cholesterol levels and smoking. For prostate cancer, the gut microbiome score was second only to baseline age in the *C*-statistic (0.623, 95% CI 0.581–0.666). After adjusting for disease-specific conventional risk factors (Extended Data Fig. 6), the effect of the gut microbiome score was significant but attenuated for incident T2D (HR = 1.20 per s.d., 95% CI 1.11–1.30, *P* = 9.13 × 10^−6^) and prostate cancer (HR 1.23 per s.d., 95% CI 1.03–1.46, *P* = 0.020); no significant effect of the gut microbiome score was found for CAD and AD. Compared with models of conventional risk factors (Fig. 2), models adding the gut microbiome score yielded a ∆*C*-statistic of 0.004 (95% CI 0–0.008) for T2D and 0.005 (95% CI −0.003 to 0.013) for prostate cancer. In the subanalysis of T2D using NMR-based glucose as an additional conventional risk factor (Extended Data Fig. 3c), the effect of the gut microbiome score was slightly attenuated (HR 1.16 per s.d., 95% CI 1.07–1.26, *P* = 5.38 × 10^−4^) and the ∆*C*-statistic yielded by adding gut microbiome score to conventional risk factors was 0.003 (95% CI −0.001 to 0.006).

### Integrating polygenic, metagenomic and conventional factors

We then investigated the combined predictive performance of PRSs, the gut microbiome and conventional risk factors of their respective diseases using Cox regression models (Table 2). Although age was the strongest individual predictor for incident CAD and prostate cancer, adding the PRS and the gut microbiome score to the age increased the *C*-statistic by 0.049 (95% CI 0.030–0.066) and 0.032 (95% CI 0.011–0.052), respectively. For T2D, adding the PRS and the gut microbiome score improved the *C*-statistic over age by 0.076 (95% CI 0.057–0.095). For incident AD, adding the PRS improved the *C*-statistic over age by 0.019 (95% CI 0.011–0.026), whereas adding the gut microbiome score did not improve the *C*-statistic. For all four diseases, the model combining disease-specific conventional risk factors, PRSs and gut microbiome scores achieved higher *C*-statistics than models based on any risk factors separately (Table 2). The combined model achieved ∆*C*-statistic over conventional risk factors of 0.024 (95% CI 0.013–0.035) for CAD, 0.014 (95% CI 0.007–0.021) for T2D, 0.017 (95% CI 0.009–0.024) for AD and 0.031 (95% CI 0.011–0.05) for prostate cancer.

**Table 2:** *C*-statistics and 95% CIs of sex-stratified Cox regression models for PRSs, gut microbiome scores and conventional risk factors

Model	Age	Age + PRS	Age + microbiome score	Age + PRS + microbiome score	CRFs	CRFs + PRS + microbiome score
Disease	*C*-statistic (95% CI)					
CAD	0.719 (0.695–0.743)	0.766 (0.742–0.789)	0.722 (0.698–0.747)	0.767 (0.744–0.791)	0.771 (0.748–0.793)	0.794 (0.772–0.817)
T2D	0.625 (0.605–0.646)	0.675 (0.654–0.695)	0.665 (0.644–0.685)	0.702 (0.681–0.722)	0.785 (0.768–0.802)	0.799 (0.783–0.816)
AD	0.880 (0.864–0.895)	0.898 (0.883–0.914)	0.880 (0.864–0.895)	0.898 (0.883–0.914)	0.883 (0.868–0.899)	0.900 (0.885–0.915)
Prostate cancer	0.769 (0.739–0.798)	0.797 (0.766–0.828)	0.774 (0.745–0.802)	0.801 (0.770–0.832)	0.773 (0.744–0.802)	0.804 (0.774–0.834)

CRFs, conventional risk factors.

The subgroup analyses for CAD, T2D and AD showed consistent results in general. In the sex-stratified Cox model for CAD (Extended Data Fig. 2d), adding the PRS and the gut microbiome score increased *C*-statistics by 0.050 (95% CI 0.030–0.068) over age and 0.025 (95% CI 0.013–0.038) over all conventional risk factors in individuals without baseline use of antihypertensives or lipid-lowering medications. For T2D (Extended Data Fig. 3d), adding the PRS and gut microbiome score improved the *C*-statistic over age by 0.073 (0.051–0.092) and the combined model increased the *C*-statistic by 0.010 (95% CI 0.003–0.016) compared with the model of conventional risk factors including NMR-based glucose. In the subgroup analysis for AD in those aged >60 years at baseline, adding the PRS improved the *C*-statistic over baseline age by 0.077 (95% CI 0.043–0.108), while the gut microbiome score did not show improvement.

In the combined models (Supplementary Tables 2–5), PRSs were found to be significantly associated with CAD (HR per s.d. 1.68, 95% CI 1.50–1.88, *P* = 4.39 × 10^−19^), T2D (HR per s.d. 1.41, 95% CI 1.29–1.54, *P* = 1.38 × 10^−14^), AD (HR per s.d. 1.93, 95% CI 1.73–2.15, *P* = 3.85 × 10^−32^) and prostate cancer (HR per s.d. 1.72, 95% CI 1.46–2.02, *P* = 1.05 × 10^−10^). The gut microbiome score was associated with T2D (HR per s.d. 1.19, 95% CI 1.10–1.29, *P* = 2.11 × 10^−5^) and prostate cancer (HR per s.d. 1.19, 95% CI 1.01–1.41, *P* = 0.041).

In subgroup analyses (Supplementary Tables 6–8), similar effects of PRSs were found for CAD (HR per s.d. 1.77, 95% CI 1.56–2.02, *P* = 3.05 × 10^−18^), T2D (HR per s.d. 1.40, 95% CI 1.27–1.53, *P* = 3.43 × 10^−12^) and AD (HR per s.d. 1.88, 1.65–2.13, *P* = 8.33 × 10^−23^); the effect of the gut microbiome score remained significant for T2D (HR per s.d. 1.15, 95% CI 1.06–1.25, *P* = 1.07 × 10^−3^) after adjusting for NMR-based glucose and other conventional risk factors.

## Discussion

While the interplay between host genetics and the gut microbiome has been increasingly recognized and studied^31,51,52^, few studies have investigated their combined impact on complex disease risk. The present study presents a joint analysis of genotyping data, gut metagenomics data and clinical metadata for four common complex diseases (CAD, T2D, AD and prostate cancer) in a large prospective population-based cohort. We compared popular published PRSs for each disease, baseline gut metagenomics and conventional risk factors for predicting the onset of each disease over a median of 17.8 years of follow-up. Our analyses reinforce the evidence that baseline age is the dominant individual risk factor for CAD, AD and prostate cancer, and adding the PRS and gut microbiome substantially improved the predictive performance to a similar capacity achieved by the combination of all conventional risk factors. We further demonstrated that PRSs improved prediction performance over the combination of conventional risk factors for all diseases studied, yet there was only mild evidence that the gut microbiome improved prediction performance when modeled jointly with conventional risk factors. The information (for example, features and coefficients) necessary to independently apply our integrated predictive models are provided in Supplementary Tables 2–5.

As expected, in our study, a higher PRS was significantly associated with higher disease incidence for all four diseases, consistent with previous studies. Also expected, we found that PRSs for all four diseases improved predictive ability over conventional risk factors, adding to the body of evidence^9,14^ that PRSs have potential clinical utility to complement traditional risk factors. Consistent with prior work, we demonstrated that PRSs improved prediction of CAD, T2D and prostate cancer independently of and in addition to family history, a strong risk factor for all diseases studied^53–57^. Notably, for AD, with the risk of development attributed to genetics being estimated at 70% (ref. ^58^), the PRS improved the *C*-statistic over conventional risk factors, including age by 0.017 in all studied participants and 0.064 in participants aged ≥60 years at baseline.

Although the ∆*C*-statistics for gut microbiome scores over conventional risk factors were small, we observed significant improvement in sex-stratified prediction models over baseline age alone for CAD, T2D and prostate cancer^26,59–61^. In accordance with previous studies, we found a significant inverse signal between baseline *α* diversity and incident T2D^62^, which could be partially explained by possible mediation effects of gut microbiota-derived metabolites correlating with lower microbial diversity (for example, imidazole propionate) and insulin resistance^63,64^. We also found significant associations between *β* diversity and incident T2D, which might indicate a shift in microbiome composition involved in disease pathogenesis and progression^26,65,66^.

Our results suggest that the physiological and metabolic processes influenced by risk-associated changes in the gut microbiome vary across diseases. For CAD and T2D, the gut microbiome score exhibited predictive performance comparable to SBP, cholesterol levels and triglycerides. For CAD, AD and prostate cancer, the microbiome score’s predictive effects were largely captured by baseline age; however, this was true to a lesser extent with T2D (Extended Data Fig. 6). The variability in the predictive capacity of the gut microbiome might be partially explained by the reciprocal relationship between host aging and microbial alterations, where age-related and disease-related changes of gut microbiota bidirectionally interact with age-related diseases such as CAD, AD and prostate cancer^67^.

Our study has limitations. First, the gut microbiome and conventional risk factors were measured only once at the initial assessment. Although the gut microbiome remains largely stable during adulthood, the microbial community is influenced by environment and cohabitation in the long term^38,68,69^; thus their effects on future disease may change from what we estimated here. In particular, the assessment of predictive capacity for the gut microbiome might be hindered by the overlapping nature of changes in the microbiome and aging-related processes that lead to disease^67^. Second, owing to unavailability, we did not assess the impact of family history of AD, a risk factor that may also capture important aspects of shared environment influencing gut microbiome composition^70,71^. Third, the generalizability of the microbiome and integrated risk models to other external cohorts could not be investigated owing to the paucity of large prospective studies with similar data types. The composition of the human gut microbiome differs across geographically and culturally distinct settings, which can be attributed to variations in host genetics, immunity and behavioral features^72,73^. Last, our study cohort comprised European ancestry (Finnish) participants; thus predictive performance of the PRS and improvement over conventional risk factors may not generalize to other demographics and healthcare systems, particularly as the predictive performance of the PRSs derived in Europeans is known to be attenuated when applied to populations of non-European ancestries^74–76^.

In summary, this work presents one of the first studies on prediction of incident common complex diseases integrating PRSs, gut metagenomics and clinical metadata. Our study highlights potential limitations in the use of the human gut microbiome for improving clinical risk prediction despite its association with incident disease; however, larger studies are warranted to better quantify potential incremental gains. Overall, we show that integrating PRSs and gut metagenomic scores can maximize predictive capacity for common diseases over conventional risk factors alone.

## Methods

### Study design

The FINRISK surveys have been conducted to investigate risk factors for major chronic noncommunicable diseases every 5 years since 1972 in Finland^77^. This work was based on the FINRISK 2002 cohort, which contains metagenome data linked to comprehensive metadata at a baseline clinical visit and prospective follow-up and has been studied for the association between gut microbiota and various health outcomes^4,26,28,29,31,78^. The study included independent and representative population samples of six geographical areas of Finland: (1) North Karelia, (2) North Savo, (3) Turku and Loimaa, (4) Helsinki and Vantaa, (5) Oulu and (6) Lapland; these were randomly drawn from the National Population Information System^47^. With an overall participant rate of 65%, the FINRISK 2002 cohort comprised a total of 8,783 individuals, including both men and woman, out of 13,498 invitees aged 25–74 years. The participants filled in self-administered questionnaires, undertook health examinations conducted by trained personnel at the study sites and donated biological samples including venous blood and stool. All participants gave written informed consent and the study protocol was approved by the Coordinating Ethics Committee of the Helsinki University Hospital District (ref. no. 558/E3/2001). The FINRISK participation was voluntary and no financial compensation was paid. The surveys were conducted in accordance with the World Medical Association’s Declaration of Helsinki on ethical principles. In the present study, we included individuals whose genotyping data and shotgun metagenomics sequencing of stool samples were both available. We excluded individuals with (1) low reads of metagenomic sequencing (total mapped reads <100,000), (2) baseline pregnancy, (3) BMI ≤40 kg m^−2^ or <16.5 kg m^−2^ and (4) antibiotic use up to 1 month before baseline. Altogether, samples from 5,676 participants were eligible for the present study.

### Baseline examination and sample collection

Demographic factors, physiological measurements, lifestyle factors, biomarkers and biological samples were collected at baseline in 2002^47^. Questionnaires and invitation to health examinations were mailed to all subjects. Self-administered questionnaires included information such as participant’s background, medical history, diet and self-reported family history of some diseases. Questionnaires were in paper form and saved to electronic format. The health examination and blood sampling were performed by trained nurses at local health centers or other survey sites. Physical measurements such as weight, height and BP were obtained during the health examination. Venous blood samples were collected for the full cohort. The samples were collected after the participants were fasted for ≥4 h and centrifuged at the field survey sites. The fresh samples were transferred daily to the central laboratory of the Finnish Institute for Health and Welfare and analyzed over the next 2 days.

Stool samples were collected from willing participants at home by using an ad hoc kit constructed in-house at the Finnish Institute for Health and Welfare with detailed instructions and a scoop method. The participants were advised to collect the sample preferably in the morning, but any time convenient to the participant was considered acceptable. The samples were mailed overnight between Monday and Thursday to the laboratory of the Finnish Institute for Health and Welfare under winter conditions in Finland and immediately stored at −20 °C on receipt to minimize potential effects of temperature on variation in microbiome composition^79^. Special care was taken to avoid delayed transit at the post office over the weekend. The sample collection was done under winter conditions with average temperatures well below 0 °C in Finland from January 2002 to March 2002, and no special arrangements were made with regard to the temperature during transportation. Although possible short-term exposure of samples to room temperature after collection may result in slight variations in the detection and relative abundances of rare taxa^80^, these variations are relatively minor considering the low environmental temperatures and the primary focus of the present study on common taxa. The stool samples were kept unthawed until 2017 when they were transferred to the University of California San Diego for sequencing.

### Disease endpoints, exclusion criteria and factors

We studied four incident diseases: CAD, T2D, AD and prostate cancer. The participants were followed up until 31 December 2019 using EHR linkage to the Finnish national registries. Disease cases were identified based on *International Classification of Diseases* (ICD)^81^ codes, Anatomical Therapeutic Chemical (ATC) codes, from the Care Register for Health Care (hospital discharges and specialized outpatient care), Finnish Cancer Register and the Drug Reimbursement and Purchase Registers. CAD cases were defined by ICD-10 I20.0|I21|I22, ICD-9 410|4110, ICD-8 410|4110; T2D cases were defined by ICD-10 E1 (refs. ^1–4^), ICD-9 250, ICD-8 250, Kela drug reimbursement code 215 and ATC A10B; AD cases were defined by ICD-10 G30|F00, ICD-9 331.0, ICD-8 290.10, Kela reimbursement code 307, reimbursement with ICD code G30|F00|3110 and ATC N06D; prostate cancer cases were identified in the Finnish Cancer Register. Follow-up time was extracted from EHRs and determined by the years to the first incident event, or death, or end of the follow-up study period.

The conventional risk factors for CAD were defined as follows: age, sex, BMI, SBP, total cholesterol, HDL-cholesterol, current smoking status, exercise, any prevalent diabetes and parental history of myocardial infarction^12^. Smoking status was defined as current use of tobacco products at baseline. Exercise was defined as regular exercise for at least 3 h per week or regular competitive sports training according to responses to self-administered questionnaires. Individuals with missing values of risk factors were excluded. Individuals with prevalent diagnosis of heart diseases were excluded. A total of 5,093 individuals were considered for CAD analyses. In the subanalysis of CAD, participants with baseline use of antihypertensives or lipid-lowering medications were further excluded, resulting in a subset of 4,293 individuals.

For T2D, the risk factors included age, sex, BMI, SBP, total cholesterol, HDL, triglycerides, current smoking status, exercise and parental history of any diabetes^26,54^. After individuals with incomplete values of risk factors, any prevalent diabetes, baseline use of diabetes medication and HbA1c (if available) ≥6.5% were excluded, a total of 5,297 individuals were involved in T2D analyses. In an additional subanalysis of T2D, baseline glucose determined by the Nightingale Health NMR platform from frozen serum samples was included as an additional risk factor in a subset of 4,911 individuals.

For AD, the risk factors included age, sex, BMI, SBP, DBP, total cholesterol, HDL, average weekly alcohol consumption, current smoking status, exercise, prevalent T2D, prevalent stroke and any prevalent psychiatric disorders including depression, bipolar disorder and schizophrenia^82^. We excluded individuals with missing values of risk factors and prevalent dementia, which resulted in 5,347 individuals for analyses of AD. The subanalysis of AD in participants aged ≥60 years at baseline included 1,220 individuals.

For prostate cancer analyses, the risk factors included age, BMI, average weekly alcohol consumption, exercise, current smoking status and parental history of any cancer^83^. Only male participants were studied. After individuals with incomplete risk factors and prevalent diagnosis of prostate cancer have been excluded, a total of 2,464 individuals remained for analyses of prostate cancer.

### Characterization of gut microbiome

DNA extraction was performed using the MagAttract PowerSoil DNA kit (QIAGEN) and the Earth Microbiome Project protocols^84^. The library generation was carried out with a miniaturized version of the Kapa HyperPlus Illumina-compatible library prep kit (Kapa Biosystems)^85^. The DNA extracts were normalized to 5 ng of total input per sample using an Echo 550 acoustic liquid-handling robot (Labcyte Inc.). Enzymatic fragmentation (1/10 scale), end-repair and adapter-ligation reactions were performed using a Mosquito HV liquid-handling robot (TTP Labtech Inc.). Sequencing adapters were based on the iTru protocol^86^, where short universal adapter stubs are ligated first followed by addition of sample-specific barcoded sequences in a subsequent PCR step. Amplified and barcoded libraries were quantified by the PicoGreen assay and sequenced on an Illumina HiSeq 4000 instrument to an average depth of ~900,000 reads per sample. The stool shotgun sequencing was successfully performed in 7,231 individuals. Adapters and low-quality sequences were trimmed with Atropos v.1.1.5 (ref. ^87^) and host reads were removed with Bowtie2 v.2.3.3 (ref. ^88^) against the human genome assembly GRCh38. The shotgun metagenomic sequences were analyzed with Oecophylla (https://github.com/biocore/oecophylla) based on Snakemake workflow^85,89^. Stool metagenomes were classified using Kraken2 v.2.1.0 (ref. ^90^) and a customized index database based on species definitions from 258,406 reference genomes (comprising 254,090 bacterial and 4,316 archaeal genomes) from GTDB release R06-RS202 (27 April 2021)^91^. Bracken v.2.5.0 (ref. ^92^) was used to re-estimate abundances after Kraken2 classification. A threshold of 250 reads per taxon was used to define a positive hit, which resulted in 4,026 species identified with a mean prevalence rate of 4.74%. After removing samples with total mapped read counts <100,000 reads per sample, taxonomic profiles from 7,205 individuals were retained for analyses with 698,067 reads per sample median depth, a minimum of 100,082 reads per sample and a maximum of 19,671,923 reads per sample.

### Genotype data processing and polygenic score calculation

Genotyping was undertaken using Illumina genome-wide SNP arrays (HumanCoreExome BeadChip, Human610-Quad BeadChip and HumanOmniExpress)^56^. After samples with ambiguous gender, missingness >5%, excess heterozygosity and non-European ancestries had been removed and variants with missingness >2%, Hardy–Weinberg equilibrium *P* < 1 × 10^−6^ and minor allele count <3 were excluded, the samples were prephased with Eagle2 v.2.3. A Finnish-population-specific reference panel consisting of 2,690 high-coverage, whole-genome sequencing and 5,092 whole-exome sequencing samples was used with IMPUTE2 v.2.3.2 to perform genotype imputation. Postimputation quality control was applied using PLINK v.2.0. Variants with INFO score <0.7, minor allele frequency <1% and Hardy–Weinberg equilibrium *P* < 1 × 10^−6^ were excluded. Samples with missing rate >10% were excluded. A total of 7,967,866 variants and 7,281 samples remained after quality control.

For all diseases studied, we calculated PRSs in the FINRISK 2002 cohort using external summary statistics in the Polygenic Score Catalog^48^. We considered previously published scores that were developed mainly based on large European populations and did not include FINRISK 2002 participants in their development. The Polygenic Score Catalog IDs of the PRSs for CAD, T2D, AD and prostate cancer were PGS000018 (ref. ^12^), PGS000036 (ref. ^42^), PGS000334 (ref. ^43^) and PGS000662 (ref. ^45^), respectively. Each PRS was computed by multiplying the genotype dosage of each risk allele at each variant by its weight and summing across all variants in the respective score with PRSice-2 (ref. ^93^). The final PRSs consisted of 1,396,966 variants for the CAD PRSs, 129,793 for the T2D PRSs, 21 for the AD PRSs and 181 for the prostate cancer PRSs.

### Statistics and reproducibility

Cox proportional hazard models stratified by sex were first fit for time on study for each incident disease on each of their respective conventional risk factors and PRSs separately. Next, a model combining disease-specific PRSs and conventional risk factors was fit for each disease. Prostate cancer was obviously studied only in men; its respective analysis did not include sex stratification. The ability of models to distinguish between cases and non-cases was assessed and compared with Harrell’s *C*-statistic, a performance metric for evaluating model discrimination based on censored survival data. Proportional hazard assumptions were examined by Schoenfeld residuals. HR, 95% CIs and two-sided Wald’s test *P* values were reported for risk factors. Statistical significance was determined with a *P*-value threshold of 0.05.

The gut microbiota diversities were measured with species-level abundance data before filtering taxa by relative abundance and prevalence. Rarefaction was not directly performed to avoid loss of data and samples had total mapped reads >100,000 after filtering. The *α* diversity of the gut microbiome was measured by Shannon’s diversity, chao1 and evenness using raw counts. As the original Shannon index can exhibit bias owing to unobserved taxa, a nearly unbiased estimator of Shannon entropy proposed by Chao et al. using subsampling taxa and extrapolation was implemented^49,94,95^. The *β* diversity was estimated separately in samples by applying PCA on centered log ratio (CLR) transformed abundance data, that is, using the Aitchison distance, after disease-specific exclusion criteria were applied. Cox proportional hazard models were fit for time on study for each disease on gut microbiome *α* diversity and the first five PCs of CLR abundance, adjusting for conventional risk factors and stratified by sex (except for prostate cancer analyses).

We subsequently focused on common and abundant taxa that were detected with a prevalence >1% and relative abundance >0.1% in at least 10% of samples. After excluding rare and less prevalent taxa, 235 species-level taxonomic groups were obtained and CLR transformed for prediction modeling. For each incident disease studied, we evaluated the predictive capacity of the gut microbiome composition using Ridge logistic regression models of species-level CLR abundance with repeated cross-validation (three-fold, repeated ten times) stratified for disease status where the training and testing data were separate in each iteration. The prevalidated predicted values in the testing sets based on the optimal cross-validated models trained on species-level CLR abundances were used as the gut microbiome scores in assessing the association between the gut microbiome and incident disease. The optimal *λ* value of Ridge models was determined from a grid search space ranging from 0.0001 to 100. The prediction performance was assessed using AUROC. For comparison, random forests were performed using repeated cross-validation with the same resampling of each iteration. Overall, random forests were outperformed by Ridge regression, with average cross-validated AUROC of 0.551 (range 0.540–0.559) for CAD, 0.570 (0.564–0.579) for T2D, 0.542 (0.531–0.560) for AD and 0.562 (0.540–0.577) for PC. For each disease studied, sex-stratified (except for prostate cancer) Cox regression model was fit for time on study on the gut microbiome score by itself and with adjustment of disease-specific conventional risk factors.

Finally, we investigated whether disease-specific PRSs and microbiome scores made independent contributions to predicting disease risk. For each incident disease, sex-stratified (except for prostate cancer) Cox models were fit on disease-specific PRSs and microbiome scores separately and in combination, adjusting for age at baseline; Cox models were also fit on baseline age alone for comparison. Sex-stratified (except for prostate cancer) Cox models were then fit on disease-specific PRSs, gut microbiome scores and conventional risk factors, and compared with Cox models combining disease-specific conventional risk factors. Covariates and their respective coefficients in Cox regression models for all diseases studied are reported in Supplementary Tables 2–8.

Statistical analysis was performed with R v.4.2.1 and v.3.6.0, R packages data.table v.1.14.2, survival v.3.2.13, compositions v.2.0.4, iNEXT v.3.0.0, otuSummary v.0.1.2, caret v.6.0.90, glmnet v.4.1.3 and v.2.0.18, boot v.1.3.28, pROC v.1.18.0, ggplot2 v.3.3.5, gridExtra v.2.3, grid v.4.1.2 and cowplot v.1.1.1. The present study is observational so randomization or blinding does not apply. The survey was a population-based study of individuals drawn from the Finnish National Population Register stratified by geographical area, sex and 10-year age group^47^. Exclusion criteria based on quality control standards, baseline characteristics of participants and disease-specific factors are detailed in Methods where relevant. Data distribution was assumed to be normal, but this was not formally tested. No statistical methods were used to predetermine sample sizes but our sample sizes are similar to those reported in previous publications^26,29,31^.

### Reporting summary

Further information on research design is available in the Nature Portfolio Reporting Summary linked to this article.

### Supplementary information

Reporting Summary

Supplementary Tables Supplementary Table 1: Cox’s regression of *α* diversities adjusting for disease-specific conventional risk factors of incident diseases. Cox’s models for incident CAD, T2D and AD are stratified by sex. *P* values for two-sided Wald’s tests. Supplementary Table 2: Sex-stratified Cox’s models of PRSs, gut microbiome scores and conventional risk factors for incident CAD. *P* values for two-sided Wald’s tests. Supplementary Table 3: Sex-stratified Cox’s models of PRSs, gut microbiome scores and conventional risk factors for incident T2D. *P* values for two-sided Wald’s tests. Supplementary Table 4: Sex-stratified Cox models of PRSs, gut microbiome scores and conventional risk factors for incident AD. *P* values for two-sided Wald’s tests. Supplementary Table 5: Sex-stratified Cox’s models of PRSs, gut microbiome scores and conventional risk factors for incident prostate cancer. *P* values for two-sided Wald’s tests. Supplementary Table 6: Sex-stratified Cox’s models of PRSs, gut microbiome scores and conventional risk factors for incident CAD in subanalysis of individuals who were not taking antihypertensives or lipid-lowering medication at baseline. *P* values for two-sided Wald’s tests. Supplementary Table 7: Sex-stratified Cox’s models of PRSs, gut microbiome scores and conventional risk factors for incident T2D in subanalysis including NMR-based glucose as an additional risk factor. *P* values for two-sided Wald’s tests. Supplementary Table 8: Sex-stratified Cox’s models of PRSs, gut microbiome scores and conventional risk factors for incident AD in subanalysis of individuals aged ≥60 years at baseline. *P* values for two-sided Wald’s tests.

### Source data

Source Data Fig. 1 Statistical source data for Fig. 1.

Source Data Fig. 2 Statistical source data for Fig. 2.

Source Data Extended Data Fig. 1 Statistical source data for Extended Data Fig. 1.

Source Data Extended Data Fig. 2 Statistical source data for Extended Data Fig. 2.

Source Data Extended Data Fig. 3 Statistical source data for Extended Data Fig. 3.

Source Data Extended Data Fig. 4 Statistical source data for Extended Data Fig. 4.

Source Data Extended Data Fig. 5 Statistical source data for Extended Data Fig. 5.

Source Data Extended Data Fig. 6 Statistical source data for Extended Data Fig. 6.

## Data Availability

The FINRISK data for the present study are available with a written application to the THL Biobank as instructed on the website of the Biobank (https://thl.fi/en/web/thl-biobank/for-researchers). A separate permission is needed from FINDATA (https://www.findata.fi/en/) for use of the EHR data. Metagenomic data are available through the European Genome–Phenome Archive (EGAD00001007035). PRSs are available through the Polygenic Score Catalog (https://www.pgscatalog.org). GTDB R06-RS202 is available through http://gtdb.ecogenomic.org. Genome assembly GRCh38 is available at http://genome.ucsc.edu. The models and statistical source data generated in the analysis are provided as Supplementary tables and source data. All other data supporting the findings of the present study are available from the corresponding author upon reasonable request.

## Code availability

The codes for the main analyses are deposited at https://github.com/dpredprj/PRS_GMS_prediction.
