# Supplementary material for: Integration of polygenic and gut metagenomic risk prediction for common diseases
Source: Nat Aging. 2024 Mar 25;4(4):584–94. doi: 10.1038/s43587-024-00590-7 (PMC11031402; doi:10.1038/s43587-024-00590-7)
Supplement: Supplementary file 1 — Reporting Summary [file 43587_2024_590_MOESM1_ESM.pdf]

nature portfolio

Corresponding author(s): Yang Liu, Michael Inouye

Last updated by author(s): Jan 9, 2024

Reporting Summary

Nature Portfolio wishes to improve the reproducibility of the work that we publish. This form provides structure for consistency and transparency in reporting. For further information on Nature Portfolio policies, see our Editorial Policies and the Editorial Policy Checklist.

Statistics

For all statistical analyses, confirm that the following items are present in the figure legend, table legend, main text, or Methods section.

n/a	Confirmed	
☐	☒	The exact sample size (*n*) for each experimental group/condition, given as a discrete number and unit of measurement
☐	☒	A statement on whether measurements were taken from distinct samples or whether the same sample was measured repeatedly
☐	☒	The statistical test(s) used AND whether they are one- or two-sided<br>*Only common tests should be described solely by name; describe more complex techniques in the Methods section.*
☐	☒	A description of all covariates tested
☐	☒	A description of any assumptions or corrections, such as tests of normality and adjustment for multiple comparisons
☐	☒	A full description of the statistical parameters including central tendency (e.g. means) or other basic estimates (e.g. regression coefficient) AND variation (e.g. standard deviation) or associated estimates of uncertainty (e.g. confidence intervals)
☐	☒	For null hypothesis testing, the test statistic (e.g. *F*, *t*, *r*) with confidence intervals, effect sizes, degrees of freedom and *P* value noted<br>*Give P values as exact values whenever suitable.*
☒	☐	For Bayesian analysis, information on the choice of priors and Markov chain Monte Carlo settings
☒	☐	For hierarchical and complex designs, identification of the appropriate level for tests and full reporting of outcomes
☐	☒	Estimates of effect sizes (e.g. Cohen's *d*, Pearson's *r*), indicating how they were calculated

Our web collection on statistics for biologists contains articles on many of the points above.

Software and code

Policy information about availability of computer code

Data collection	n/a
Data analysis	Sequencing was performed on Illumina HiSeq 4000 platform with Kapa HyperPlus kits, following the previously published protocol (https://doi.org/10.1186/s13059-019-1834-9). Adapters and low-quality sequences were trimmed with Atropos v1.1.5, and host reads were removed with Bowtie2 v2.3.3 against the human genome assembly GRCh38. Taxonomic profiling was conducted with Kraken2 v2.1.0 and Genome Taxonomy Database (https://gtdb.ecogenomic.org/) release R06-RS202. Bracken v2.5.0 was used to re-estimate abundances after Kraken2 classification. A Finnish population-specific reference panel was used with IMPUTE2 v2.3.2 to perform genotype imputation. Post-imputation quality control was applied using PLINK v.2.0. Polygenic risk scores were calculated using external summary statistics in the Polygenic Score Catalog with PRSice-2. The codes for main analyses are deposited at https://github.com/dpredprj/PRS_GMS_prediction. Statistical analysis was performed with R versions 4.2.1 and 3.6.0. R packages: data.table 1.14.2, survival 3.2.13, compositions 2.0.4, iNEXT3.0.0, otuSummary 0.1.2, caret 6.0.90, glmnet 4.1.3 and 2.0.18, boot 1.3.28, pROC 1.18.0, ggplot2 3.3.5, gridExtra 2.3, grid 4.1.2, cowplot 1.1.1

For manuscripts utilizing custom algorithms or software that are central to the research but not yet described in published literature, software must be made available to editors and reviewers. We strongly encourage code deposition in a community repository (e.g. GitHub). See the Nature Portfolio guidelines for submitting code & software for further information.

Data

Policy information about availability of data

All manuscripts must include a data availability statement. This statement should provide the following information, where applicable:

- Accession codes, unique identifiers, or web links for publicly available datasets
- A description of any restrictions on data availability
- For clinical datasets or third party data, please ensure that the statement adheres to our policy

The FINRISK data for the present study are available with a written application to the THL Biobank as instructed on the website of the Biobank (https://thl.fi/en/web/thl-biobank/for-researchers). A separate permission is needed from FINDATA (https://www.findata.fi/en/) for use of the EHR data. Metagenomic data are available through the European Genome-Phenome Archive (EGAD00001007035). PRSs are available through PGS Catalog (https://www.pgscatalog.org/). GTDB R06-RS202 is available through http://gtdb.ecogenomic.org. Genome assembly GRCh38 is available via http://genome.ucsc.edu. The models and statistical source data generated in the analysis are provided as Supplementary Data and Tables.

Research involving human participants, their data, or biological material

Policy information about studies with human participants or human data. See also policy information about sex, gender (identity/presentation), and sexual orientation and race, ethnicity and racism.

Reporting on sex and gender	The term "sex" was used and was determined based on self-reporting. Sex-stratified Cox regression analysis was performed for incident coronary artery disease, type 2 diabetes and Alzheimer's disease. Analyses for prostate cancer applied to only male sex.
Reporting on race, ethnicity, or other socially relevant groupings	Socially relevant variables including baseline age, family history, lifestyle factors and prevalent diseases were based on self-reporting and linked electronic health registers. Definitions of these variables were detailed in the Methods section in this study. Social factors were used as a covariate in statistical models.
Population characteristics	The FINRISK 2002 study was based on a stratified random sample of the population aged 25–74 years from six specific geographical areas of Finland. Covariate-relevant characteristics include demographic, anthropomorphic, lifestyle factors, disease-specific clinical laboratory measurements, family history and diagnoses of prevalent diseases. Details of the participants' characteristics are summarized in Table 1 and the Methods section.
Recruitment	The FINRISK surveys have been conducted to investigate risk factors for major chronic non-communicable diseases every 5 years since 1972 in Finland, and this work was based on FINRISK study carried out in 2002. The study included independent and representative population samples of six geographical areas of Finland: (1) North Karelia, (2) Northern Savo, (3) Turku and Loimaa, (4) Helsinki and Vantaa, (5) Oulu and (6) Lapland, that were randomly drawn from the Finnish National Population Information System. With an overall participant rate of 65%, the FINRISK 2002 cohort comprised a total of 8,783 individuals out of 13,498 invitees.
Ethics oversight	All participants gave written informed consent, and the study protocol was approved by the Coordinating Ethics Committee of the Helsinki University Hospital District (Ref. 558/E3/2001). The FINRISK participation was voluntary and no financial compensation was paid.

Note that full information on the approval of the study protocol must also be provided in the manuscript.

Field-specific reporting

Please select the one below that is the best fit for your research. If you are not sure, read the appropriate sections before making your selection.

☒ Life sciences ☐ Behavioural & social sciences ☐ Ecological, evolutionary & environmental sciences

For a reference copy of the document with all sections, see nature.com/documents/nr-reporting-summary-flat.pdf

Life sciences study design

All studies must disclose on these points even when the disclosure is negative.

Sample size	The FINRISK 2002 study was a population study. The samples were representative of the Finnish population and were among the largest cohorts with metagenomic sequencing. No statistical methods were used to pre-determine sample sizes but our sample sizes are similar to those reported in previous publications (https://doi.org/10.1038/s41588-021-00991-z, https://doi.org/10.1038/s41467-021-22962-y, DOI: 10.2337/dc21-2358). In the present study, we included individuals whose genotyping data and shotgun metagenomics sequencing of stool samples were both available. Altogether, samples from 5,676 participants were eligible for this study. After disease-specific exclusion criteria were applied: CAD n= 5,093; T2D n= 5,297; AD n= 5,347; and prostate cancer n= 2,464. Sub-analyses of CAD n=4,293, T2D n=4,911, Alzheimer’s disease n=1,220.
Data exclusions	Individuals with low reads of metagenomic sequencing (total mapped reads <100,000), baseline pregnancy, baseline BMI>=40 kg/m2 or <16.5 kg/m2, antibiotic use up to one month prior to baseline, or missing values of risk factors were excluded. Disease-specific exclusion criteria were also applied. For CAD analysis, individuals with prevalent diagnosis of heart diseases were excluded, and individuals with baseline use of antihypertensives or lipid-lowering medications were further excluded in the subanalysis. For T2D analysis, individuals with any prevalent

diabetes, baseline use of diabetes medication, and glycated haemoglobin (HbA1c) (if available) >= 6.5% were excluded. For Alzheimer's disease, individuals with prevalent dementia were excluded, and individuals aged below 60 at baseline were further excluded in the subanalysis. For prostate cancer analyses, only male participants were studied and individuals with prevalent diagnosis of prostate cancer were excluded.

Replication	Experimental replication was not formally attempted. Repeated cross-validation was performed to assess variability.
Randomization	There were no intervention or experimental groups.
Blinding	During the recruitment, samples were randomly drawn from the National Population Information System in Finland. Samples were allocated to disease cases or healthy controls according to hospital diagnosis. The investigators of this study were blinded to recruitment of samples and diagnosis process.

Reporting for specific materials, systems and methods

We require information from authors about some types of materials, experimental systems and methods used in many studies. Here, indicate whether each material, system or method listed is relevant to your study. If you are not sure if a list item applies to your research, read the appropriate section before selecting a response.

Materials & experimental systems

n/a	Involved in the study	
☒	☐	Antibodies
☒	☐	Eukaryotic cell lines
☒	☐	Palaeontology and archaeology
☒	☐	Animals and other organisms
☒	☐	Clinical data
☒	☐	Dual use research of concern
☒	☐	Plants

Methods

n/a	Involved in the study	
☒	☐	ChIP-seq
☒	☐	Flow cytometry
☒	☐	MRI-based neuroimaging

Plants

Seed stocks	*Report on the source of all seed stocks or other plant material used. If applicable, state the seed stock centre and catalogue number. If plant specimens were collected from the field, describe the collection location, date and sampling procedures.*
Novel plant genotypes	*Describe the methods by which all novel plant genotypes were produced. This includes those generated by transgenic approaches, gene editing, chemical/radiation-based mutagenesis and hybridization. For transgenic lines, describe the transformation method, the number of independent lines analyzed and the generation upon which experiments were performed. For gene-edited lines, describe the editor used, the endogenous sequence targeted for editing, the targeting guide RNA sequence (if applicable) and how the editor was applied.*
Authentication	*Describe any authentication procedures for each seed stock used or novel genotype generated. Describe any experiments used to assess the effect of a mutation and, where applicable, how potential secondary effects (e.g. second site T-DNA insertions, mosiacism, off-target gene editing) were examined.*
